# Ultrasound elastography as an objective diagnostic measurement tool for lymphoedema of the treated breast in breast cancer patients following breast conserving surgery and radiotherapy

**DOI:** 10.2478/v10019-012-0033-z

**Published:** 2012-11-09

**Authors:** Nele Adriaenssens, Dries Belsack, Ronald Buyl, Leonardo Ruggiero, Catherine Breucq, Johan De Mey, Pierre Lievens, Jan Lamote

**Affiliations:** 1 Physical Therapy Department, Universitair Ziekenhuis Brussel, Belgium; 2 Department of Radiology, Universitair Ziekenhuis Brussel, Belgium; 3 Physical Therapy Department, Vrije Universiteit Brussel, Belgium; 4 Department of Biostatistics and Medical Informatics, Vrije Universiteit Brussel, Belgium; 5 Department of Engineering, Vrije Universiteit Brussel, Belgium; 6 Department of Oncological Surgery, Universitair Ziekenhuis Brussel, Belgium

**Keywords:** early breast cancer, breast conserving surgery, breast irradiation, lymphoedema of the breast, breast oedema, ultrasound elastography

## Abstract

**Background.:**

Lymphoedema of the operated and irradiated breast is a common complication following early breast cancer treatment. There is no consensus on objective diagnostic criteria and standard measurement tools. This study investigates the use of ultrasound elastography as an objective quantitative measurement tool for the diagnosis of parenchymal breast oedema.

**Patients and methods.:**

The elasticity ratio of the subcutis, measured with ultrasound elastography, was compared with high-frequency ultrasound parameters and subjective symptoms in twenty patients, bilaterally, prior to and following breast conserving surgery and breast irradiation.

**Results.:**

Elasticity ratio of the subcutis of the operated breast following radiation therapy increased in 88.9% of patients, was significantly higher than prior to surgery, unlike the non operated breast and significantly higher than the non operated breast, unlike preoperative results. These results were significantly correlated with visibility of the echogenic line, measured with high-frequency ultrasound. Big preoperative bra cup size was a significant risk factor for the development of breast oedema.

**Conclusions.:**

Ultrasound elastography is an objective quantitative measurement tool for the diagnosis of parenchymal breast oedema, in combination with other objective diagnostic criteria. Further research with longer follow-up and more patients is necessary to confirm our findings.

## Introduction

The National Institutes of Health Consensus Development Conference on Treatment of Early Stage Breast Cancer indicated, 22 years ago, that breast conserving surgery (BCS) with radiation therapy (RT) is the primary treatment for the majority of women with early stage breast cancer.^1^ Nowadays, BCS is, after the diagnostic procedures, the most widely used surgical procedure for early breast cancer and in most cases it is followed by whole-breast irradiation of 55–60 Gy administered in a fractionated dose over the course of five to six weeks.^2^–^5^

An adverse side effect of this treatment is breast cancer related lymphoedema of the operated and irradiated breast (breast oedema).^6^ Breast oedema is largely underdiagnosed in clinical practice because of lack of consensus on objective diagnostic criteria and on standard measurement tools.^7^,^8^ Therefore, incidence intervals are wide and incidence is strongly influenced by follow-up time and the presence of patient and therapy related risk factors.^1^,^3^ ^6^,^7^,^9^–^14^ Incidences vary between 5% and 80%.^3^,^6^,^7^,^9^–^12^

The onset of breast oedema can occur postoperatively by disturbance of the lymphatic circulation, but it is most commonly reported following breast RT, which has been blamed for increasing the incidence following BCS.^1^,^6^,^7^,^9^,^11^,^12^ RT of ≥ 40Gy may lead to a significant increase in breast volume due to tissue reactions with an oedema, but breast irradiation itself does not initiate cutaneous oedema unless other predisposing or aggravating factors are present.^13^,^14^ Breast irradiation does cause sclerosis of the skin, late post RT. Tissue fibrosis may obstruct lymph flow and slow down regeneration and formation of new lymph vessels.^15^ The time course of cutaneous breast oedema during and following RT was previously described by Wratten *et al.* in 2007.^14^ Lymph node irradiation in the breast area may decrease filter function and inhibit normal lymphatic proliferative response to inflammatory stimuli.^7^

Although pathophysiology of breast oedema is well known, problems with differential diagnosis occur frequently in clinical practice, because diagnosis and severity are quantified using subjective grading assessments by care takers and patients.^6^,^9^,^16^ The clinical presentation of breast oedema becomes present in the second phase only, when the breast volume difference is obvious with symptoms of *peau d’orange*, redness, pain and positive pitting.^3^,^9^,^12^

Objectivities of clinical symptoms with medical imaging has shown that magnetic resonance imaging (MRI) and high-frequency ultrasound (HFUS) are feasible for some criteria of breast oedema.^11^,^17^ There is a close correspondence between breast oedema demonstrated on MRI and the severity of clinical induration (palpable hardness).^17^ Breast oedema on HFUS is presented by thickening of the skin over 2 mm with increased echogenicity, disturbance or poor visibility of the deeper echogenic line and interstitial fluid accumulation.^11^ However, HFUS is not useful in quantifying parenchymal breast oedema and acute inflammatory changes induced by breast irradiation.^6^,^14^ Absolute HFUS echogenicity measures and visibility of the boundary of the dermis are strongly dependent on HFUS unit’s gain settings and therefore unusable as a quantitative measure.^6^ Since quantification at the onset of complications is necessary to evaluate the further evolution and treatment, a quantitative diagnostic measurement tool is necessary for breast oedema.

US elastography is a clinical application, non invasive, cost effective, safe and widely-accessible, that could give more information than HFUS on operation and radiation induced changes to the skin and subcutaneous tissue.^16^ US elastography is a technique used for assessment of tissue elasticity, a soft tissue characterization based on the elastic properties. The principle of sono-elastography is that tissue compression results in a displacement or strain of the tissue. This strain is lower in hard tissue compared to soft tissue. By comparing the tissue during compression and decompression, information about the hardness of tissue can be assessed using a cross-correlation technique to determine the amount of displacement of each B-mode image pixel. There are several elastography techniques, the most studied in the literature being static elastography. Static elastography technique involves obtaining US signals from an axial imaging plane prior to and following a slight compression of the tissue. Typically, the pre and post compression frames are processed to generate images of local strain, commonly known as elastograms, displayed as an elasticity colour map image. On the elastogram, strain values of the different tissues viewed on the colour map image can be compared for quantification of a strain or elasticity ratio between tissues with different elasticity.^18^–^21^ The breast is an application of choice for this technique, since it is readily accessible to compression with an US transducer.

Numerous groups have studied the elasticity of focal lesions in comparison with normal surrounding tissue within the breast, thyroid tissue, prostate and liver.^18^,^22^–^24^ Also elasticity of lymph nodes has been studied and there have been studies about the applications of elastography within musculoskeletal disciplines.^20^,^21^ Likewise, we assume that interstitial fluid accumulation in the breast following BCS with RT will affect the elasticity of subcutaneous breast tissue and therefore increase the tissues strain values.

Prior to embarking on a prospective study involving sequential observations in a large number of patients, we conducted a pilot study to assess the usefulness of US elastography to quantify parenchymal breast oedema post irradiation. The aim was to determine the correlation between qualitative changes and quantitative data in the treated breast. Main research questions are: ‘Is this quantitative technique able to measure early breast oedema in the operated and irradiated breast, as compared to the untreated contralateral breast and as compared to the preoperative breast?’ and ‘Does this quantitative technique correlate with subjective breast oedema and HFUS?’

## Patients and methods

### Patients

The study population was composed of women who were diagnosed with a primary breast cancer and scheduled for breast surgery at the University Hospital of Brussels. The trial, which was approved by the institutional ethics board, recruited women aged 18 years or older, with pathological nodal status assessed by axillary lymph node dissection (ALND) or sentinel lymph node dissection (SLND). The breast tumours have been completely resected by BCS. Enrolment into the trial took place between January 10 2011 and July 10 2011; for this report we included all evaluable patients who were measured at least twice between the trial start date and November 10 2011. The observation times were grouped to the closest planned follow-up interval. Women provided written informed consent prior to surgery. The trial was in accordance with the Helsinki Declaration of 1975, as revised in 2000.

RT was delivered as a dose of 50 Gy in 25 fractions over 5 weeks to the chest wall using tangential photon fields and to the supraclavicular, infraclavicular and axillary nodes in the case of pN1 status using an anterior field matched to the tangential fields. The majority of patients (75%) received an additional sequential boost of 16 Gy in 8 fractions over 2 weeks to the initial tumour bed using a direct electron field.^25^ One patient participated in an experimental RT design and was delivered a dose of 42 Gy in 15 fractions over 3 weeks to the whole breast and to the supraclavicular, infraclavicular and axillary nodes because of pN1 status, using the Image Guided Radiation Therapy system Tomotherapy^®^. She received a simultaneous integrated boost of 9 Gy with the 15 fractions.^26^

### Anthropomorphometric assessment

Assessments were made by a single physical therapist (NA) prior to BCS (baseline evaluation) and following completion of RT (follow-up). In this way, subjects acted as their own controls, where baseline measurements could be compared with results following RT. An assessment was also conducted prior to starting RT and is planned at six months, one year and two years post operatively, but this is not included in this report.

Baseline patient characteristics and clinical data recorded during baseline assessment were the patient’s age, weight and height, the breast that is going to be operated, localization of the tumours in the breast and the dominant side. Adjuvant treatment and type of surgery were collected from the medical file.

Breast volume was calculated from anthropomorphometric measurements on both breasts, with the formula of Qiao *et al*. described in 1997.^27^ This method was proved to be adequate, most convenient and the best method for breast volume calculation by patients and doctors. The measurements have an acceptable degree of accuracy and reproducibility, but the composition of the mathematical formula is discussable.^28^–^30^

For the physical assessment of breast cancer related lymphoedema of the arm (BCRL), we measured the volume of both arms of each patient with a mobile infrared optoelectronic volumeter (Perometer^®^ 1000M, Pero-System GmbH, Wuppertal, Germany; Peroplus Software TM). The presence of BCRL was defined as ≥10% inter-limb discrepancies in volume from baseline, respectively, where inter-limb discrepancy was computed as per cent volume difference (percentage volume difference = 100 * [volume of affected arm - volume of unaffected arm] / [volume of unaffected arm]).

Obesity was expressed as a body mass index (BMI) of ≥ 30 kg/m^2^.^7^,^9^,^11^ Preoperative bra cup size was calculated as the difference between the over-bust circumference and the underband circumference. If the difference was <6.5 cm; 6.5–13 cm; 13–19.5 or >19.5, the patient had, respectively an A, B, C or ≥D cup.^31^

### Subjective symptoms assessment

Subjective breast oedema was registered by the patient at follow-up, as an answer to a question (‘*Was the area of your operated breast swollen during the past week?*’) of the EORTC QLQ BR23 questionnaire. This questionnaire uses the 4-point categorical scale according to the modified system of Johansen *et al*.^3^ If the patient answered 1 (‘*absolutely not’*) to this question, she had no subjective breast oedema. If she answered 2, 3 or 4, she had subjective breast oedema.

Toxicity of the breast skin was scored by an experienced breast radiotherapist following the last RT session, using Radiation Therapy Oncology Group (RTOG) acute morbidity scoring schemas.^16^,^32^

### High-frequency ultrasound assessments

**FIGURE 1:**
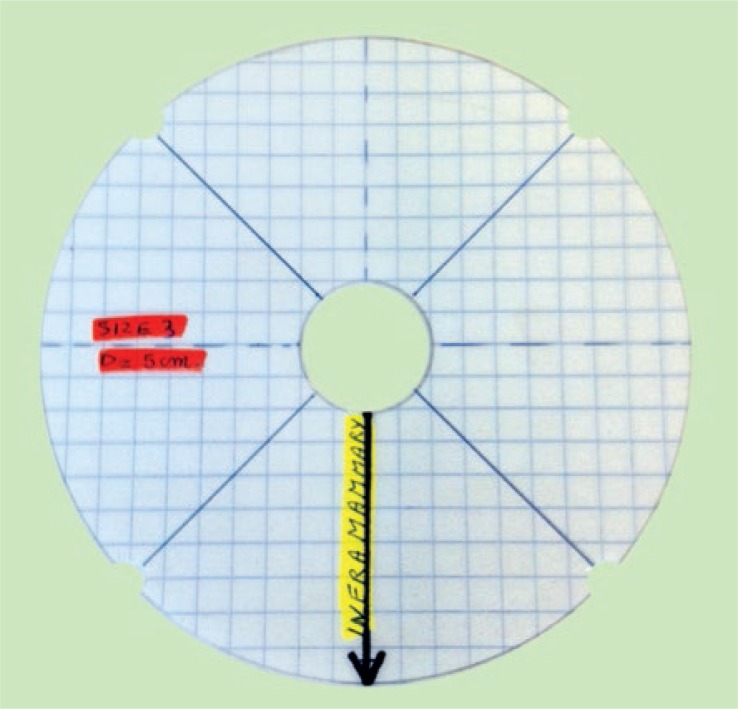
An example of a breast gauge.

HFUS evaluation of the breasts was performed using a clinical ultrasound scanner (Toshiba Aplio XG) with a High-Resolution 12-MHz linear probe (PLT 1204 BT, Toshiba) for data acquisition. The examinations were performed with patients in supine position. B-mode images were obtained focusing on the areas of interest, being the four quadrants of both breasts (SIQ, upper inner quadrant; IIQ, lower inner quadrant; IEQ, lower outer quadrant and SEQ, upper outer quadrant). To obtain the images at the same location during follow-up, plastic breast gauges of variable sizes were created and used on every patient, depending on the size of the patient’s preoperative breast cup size (Figure 1).

**FIGURE 2:**
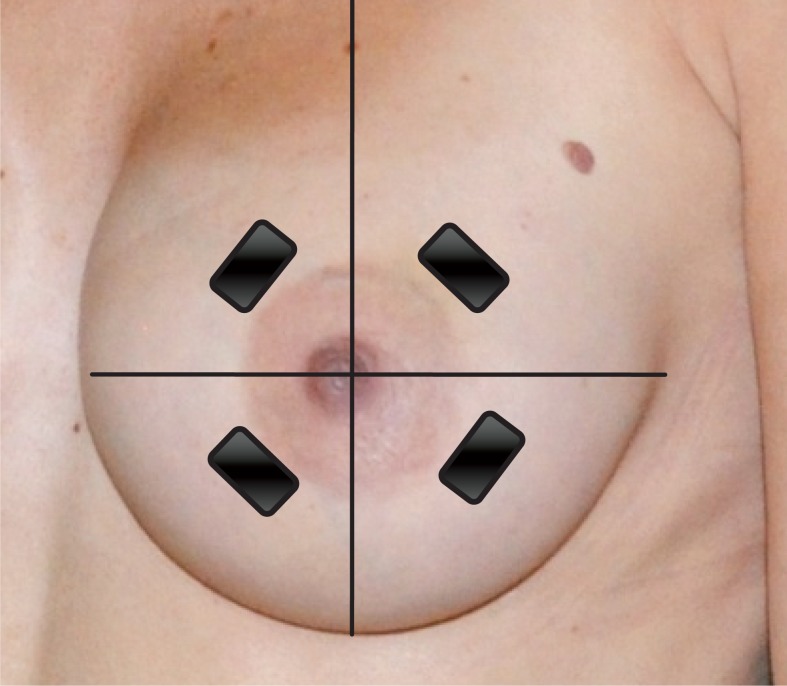
Position of the ultrasound probe in the four quadrants and the inframammary fold of the breast, perpendicular to the nipple.

To ensure good coupling of the probe-skin interface, a layer of ultrasound transmission gel was used in addition to a gel pad, SonarAid (Geistlich Pharma AG) size 130 x 120 x 10 mm. The gel pad was used as a reference tissue for elasticity measurements. The probe was held perpendicular to the breast surface, parallel with the concentric circles contouring the areola at the level indicated by the breast gauge (Figure 2). No additional compression was performed for the B-mode images. The viewing field depth was standardized at 4 cm, and the gain was not adjusted (fabric default setting, 2DG: 81). Scans were performed at the same days as the clinical assessments. HFUS examination of the breasts was performed by an experienced breast radiologist (DB). On the B-mode HFUS images, the radiologist evaluated the visibility of the deeper echogenic line and the presence of interstitial fluid accumulation between the lobules of subcutaneous fat tissue. In addition, skin thickness was calculated at every measure location of both breasts. All images were stored for later analysis. Measurements taken from the four quadrants of the operated breast were averaged for each parameter and compared with the average value of the non operated breast of the same patient and the baseline measurements of the same breast. Although radiation dose was not uniform across the entire breast, average values were analysed to validate the viability of our technique.^16^

#### Skin thickness

Skin thickening is a well-known post irradiation effect, which has been researched by several groups using HFUS. Skin thickness was measured as the distance between two thin echogenic lines with the hypoechoic dermis within, as described extensively in previous studies.^6^,^11^,^14^,^16^,^33^ Skin thickness of the four quadrants of the breast was recorded for the operated and non operated breast. Thickness of normal breast skin varies between 1 and 2 mm with a mean thickness of 1.7mm.^11^ The underlying physics concept behind skin thickness measurements has been described in previous reports.^16^

#### Echogenicity of the subcutis

IQ-VIEW/IQ-LITE, IMAGE Information Systems Ltd. 2008 v.2.5.0. R1 was used to measure the density of the subcutis in a selected region of interest (ROI). A rectangle is drawn over the specified area, with the tools application ‘measure ROI density’, and the mean, minimum, maximum and standard deviation density is given on a scale between 0 (black) and 230 (white) INT.^14^,^33^

#### Visibility of the deeper echogenic line

Evaluation of the deeper echogenic line as a marker for subcutaneous oedema, previously described by the Rönkä group, was performed by scoring the visibility of the subcutaneous fat interface between 0 (not visible) and 4 (clearly visible).^11^

#### Interstitial fluid accumulation

Presence of interstitial fluid accumulation was assessed on the B-mode US images as hypoechoic fluid infiltration (dark bands) between the fat lobules and scored as absent (score 0) or present (score 1).^11^

### Ultrasound elastography

US elastography measurements were performed following the HFUS B-mode scans at the same measurement locations in the four quadrants of both breasts as previously described, using the same conventional linear 12-MHz transducer. In our study protocol, the static US elastography technique was applied using Toshiba ElastoQ software. Given the diffuse distribution of parenchymal breast oedema, affecting large regions or even the whole breast, comparison with normal, non affected breast tissue at the same US elastography image is impossible. Therefore a gel pad was placed on the breast skin in each patient as a reference tissue for the underlying breast tissue. The elasticity of the gel pad did not change during the term of the study, making it an excellent reference tissue. The same image settings as in the HFUS B-mode images were used. For obtaining the real-time freehand US elastography images, the transducer was compressed and released perpendicular to the skin for approximately five times along the radiation axis, in the US elastography mode.

**FIGURE 3:**
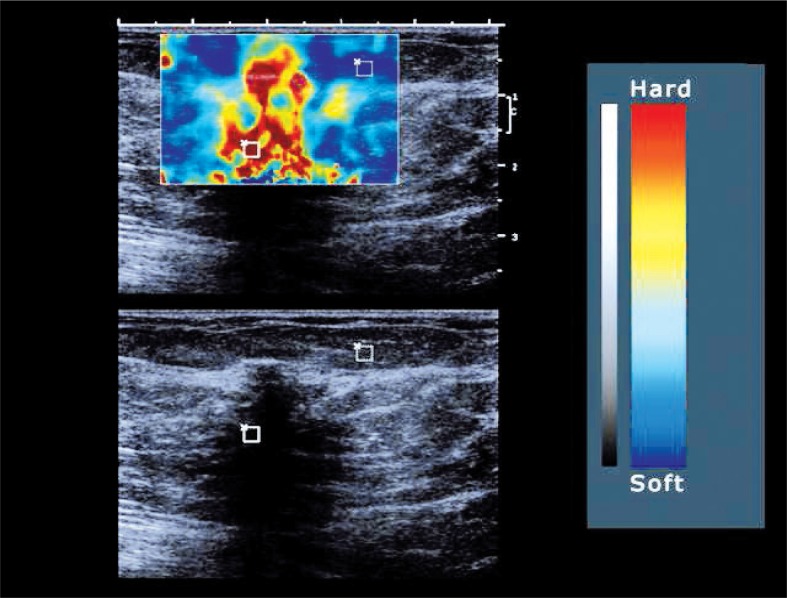
Elastogram with a region of interest (ROI) in two tissues with different elasticity.

**FIGURE 4:**
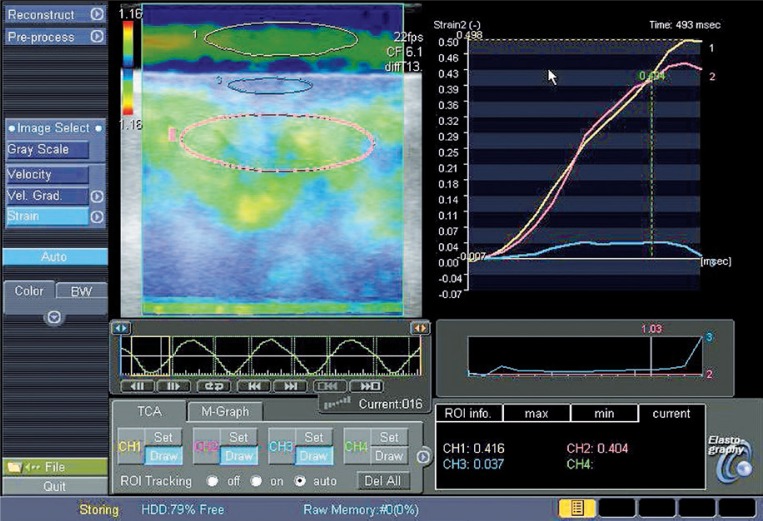
The elastogram with 3 ROI’s (yellow, gel pad; blue, skin; pink, subcutis) and the strain graph with three matching curves. The strain values are displayed underneath, respectively 0.416, 0.037, 0.404. Images were taken in the IIQ of an operated breast.

US Elastography software measured the strain image or elastogram on which quantitative strain values were assessed. On the elastogram a strain colour scale image and strain graph are displayed. ROIs were placed on the colour scale image for comparison of strain values of tissues with different elasticity (Figure 3). In our protocol, a standardized ROI box was placed in the reference gel pad (ROI length 10±1 mm, depth 4±1 mm) and in the subcutaneous breast tissue (ROI length 20±1 mm, depth 5±1 mm). From these ROIs the average strain values were displayed as curves in a strain graph for each amount of compression force during the compression/decompression cycle. The range of strain value derived from the gel pad curve during compression/decompression varied between 0 and 0.7 on average during preliminary test exams, depending on the amount of compression given. For standardization, all measurements were obtained at 0.4±0.1 strain value of the reference gel pad. The strain value of the gel pad (0.4±0.1) was divided by the strain value of the subcutaneous breast tissue at the same amount of compression, extrapolated from the gel pad value on the strain graph, giving a calculated strain ratio. These ratios were calculated in the different quadrants of each breast prior to surgery and following RT, except for the operated quadrant, because compression in this region could be painful following surgery (Figure 4).

### Statistical analysis

Data were verified on a case-by-case basis to identify inconsistencies. Descriptive statistics as frequencies, means, standard deviations and percentages were used to describe patients’ characteristics. We have used dichotomous breast oedema criteria as well as mean continuous absolute values of the four breast quadrants and single continuous absolute values of the different breast quadrants. For univariate comparisons of baseline and follow-up measurements the matched paired t-test was used. An independent t-test was performed to examine the difference between operated and non operated breast measurements.

Correlations between the breast oedema criteria were assessed using Pearson’s and Spearman Rank correlation coefficients. Pearson-Chi Square tests were used to examine the significance of the correlation between the different diagnostic criteria for breast oedema and elasticity ratio of the subcutis of the operated and non operated breast at baseline and of the operated breast following RT.

Influence of risk factors on absolute elasticity ratio of the subcutis in the operated breast following RT was assessed using a general linear model.

For all analyses, superiority was based on two-sided p values <0.05. All statistical computations used SPSS v. 20.0. (IBM Corporation, Somers, NY 10589, USA).

## Results

Of the twenty nine patients who were recruited in the trial between January 10, 2011 and September 10, 2011, twenty five received baseline evaluation and first follow-up, prior to RT (data not shown). One patient was excluded at first follow-up, because she did not have to receive RT. Four patients did not finish RT and thus second follow-up (following RT), leaving twenty patients available for analyses at November 10, 2011.

Table 1 summarizes the baseline characteristics and clinical data of the evaluable patients. The inter-limb PVD at baseline was close to zero in all patients, indicating that there were no patients with BCRL prior to and following BCS and RT (data not shown).

### Subjective swelling of the operated and irradiated breast

Subjective swelling of the operated and irradiated breast was present in 43.8% of the patients.

### Skin thickness

Mean skin thickness of the four quadrants following RT was over 2 mm in every patient. In the different quadrants, skin thickness over 2 mm was present in 90%, 90%, 80% and 75% of the patients in the SIQ, IIQ, IEQ and SEQ, respectively.

Absolute skin thickness significantly increased (p=0.000) in all quadrants of the operated breast following RT, compared with baseline measurements. In the non operated breast, absolute skin thickness did not change significantly in the quadrants.

Absolute skin thickness was significantly higher (p=0.000) in all quadrants of the operated breast following RT, compared with all quadrants of the non operated breast following RT. Baseline measurements of absolute skin thickness did not differ significantly between the operated and the non operated breast in all quadrants prior to surgery (Table 2).

### Echogenicity of the subcutis

Mean echogenicity of the subcutis in the four quadrants following RT increased in 89.5% of the patients. In the different quadrants, echogenicity of the subcutis increased in 78.9%, 70%, 80% and 61.5% of the patients in the SIQ, IIQ, IEQ and SEQ, respectively.

Absolute echogenicity of the subcutis significantly increased (p≤0.05) in all quadrants, except for the SEQ, of the operated breast following RT, compared with baseline measurements. In the non operated breast, absolute echogenicity of the subcutis did not change significantly in the quadrants.

Absolute echogenicity of the subcutis was significantly higher (p=0.000) in all quadrants of the operated breast following RT, compared with all quadrants of the non operated breast following RT. Baseline measurements of absolute echogenicity of the subcutis did not differ significantly between the operated and the non operated breast in all quadrants prior to surgery (Table 3).

### Visibility of the echogenic line

Mean visibility of the echogenic line in the four quadrants following RT decreased in every patient. In the different quadrants, visibility of the echogenic line was present in 95%, 100%, 100% and 100% of the patients in the SIQ, IIQ, IEQ and SEQ, respectively.

Mean visibility of the echogenic line significantly decreased (p=0.000) in all quadrants of the operated breast following RT, compared with baseline measurements. In the non operated breast, visibility of the echogenic line did not change significantly in the quadrants.

Mean visibility of the echogenic line was significantly lower (p=0.000) in all quadrants of the operated breast following RT, compared with all quadrants of the non operated breast following RT. Baseline measurements of visibility of the echogenic line did not differ significantly between the operated and the non operated breast in all quadrants prior to surgery.

### Interstitial fluid accumulation

Mean interstitial fluid accumulation in the four quadrants following RT increased in 72.2% of the patients. In the different quadrants, interstitial fluid accumulation was present in 83.3%, 77.8%, 77.8% and 88.9% of the patients in the SIQ, IIQ, IEQ and SEQ, respectively.

Mean interstitial fluid accumulation in the subcutis of the operated breast significantly increased (p<0.05) following RT, compared with the mean baseline measurement. Although interstitial fluid accumulation of the subcutis increased in the different quadrants, they did not increase significantly for SIQ and SEQ, only for IIQ (p=0.042) and IEQ (p=0.042). In the non operated breast, interstitial fluid accumulation of the subcutis did not change significantly in the quadrants or as a mean interstitial fluid accumulation of the subcutis in the four quadrants. Mean interstitial fluid accumulation was significantly higher (p<0.05) in all quadrants of the operated breast following RT, compared with all quadrants of the non operated breast following RT, except for SIQ and SEQ. Baseline measurements of interstitial fluid accumulation did not differ significantly between the operated and the non operated breast in all quadrants prior to surgery.

### Elasticity ratio of the subcutis

Mean elasticity ratio of the subcutis in the four quadrants following RT increased in 88.9% of the patients. In the different quadrants, elasticity ratio of the subcutis increased in 63.2%, 65%, 60% and 78.6% of the patients in the SIQ, IIQ, IEQ and SEQ, respectively.

Mean absolute elasticity ratio of the subcutis significantly increased (p<0.05) in the operated breast following RT, compared with the mean baseline measurement. Although absolute elasticity ratio of the subcutis increased in the different quadrants, they did not increase significantly. In the non operated breast, absolute elasticity ratio of the subcutis did not change significantly in the quadrants or as a mean absolute elasticity ratio of the subcutis of the four quadrants.

Mean absolute elasticity ratio of the subcutis was significantly higher (p=0.000) in the operated breast following RT, compared with the non operated breast following RT. This difference was not significantly higher for all quadrants, only for IEQ (p=0.032). Baseline measurements of absolute elasticity ratio of the subcutis did not differ significantly between the operated and the non operated breast in all quadrants prior to surgery (Table 4).

### Correlation between elasticity ratio of the subcutis and the other breast oedema criteria

A bivariate correlation between the elasticity ratio of the subcutis and the different diagnostic criteria for breast oedema, prior to surgery, in the operated and non operated breast neither showed any correlation between the variables, nor for the operated breast following RT, except for the mean visibility of the echogenic line.

### Risk factors

ALND, tumours located in the SEQ, preoperative obesity, chemotherapy (except for IEQ; more increase in the patients with chemotherapy), RT dose of 66 Gy, operated at the dominant side, age, BMI and time between surgery and start of RT, were not significant risk factors, neither for increase of absolute elasticity ratio of the subcutis, nor for increase of the elasticity ratio of the subcutis in the operated breast following RT. Bigger preoperative bra cup size was a significant risk factor for the increase of US elasticity ratio of the subcutis (p=0.01).

## Discussion

In this study, we have measured different currently used breast oedema criteria and evaluated a new objective diagnostic measurement tool for parenchymal breast oedema, prior to BCS and following RT. HFUS images and US elastography images were obtained in four quadrants of both the operated and non operated breast of 20 patients. We have compared baseline results with follow-up measurements and the operated with the non operated breast. Possible risk factors and correlations between breast oedema criteria have been investigated.

*Subjective swelling* of the operated breast, rated by the patient, was present in almost half of the patients following RT. Subjective breast oedema following RT, rated by an experienced breast radiotherapist and physical therapist (NA), resulted in respectively at least grade one skin toxicity and clinical breast oedema in all patients.^3^,^9^,^12^,^32^ Subjective symptom rating by patients and clinicians are not satisfying for the diagnosis and degree of parenchymal breast oedema.

*Skin thickness* of the operated breast following RT was over 2 mm in all patients and significantly thicker than prior to surgery, unlike the non operated breast skin. Skin thickness of the operated breast following RT was significantly thicker than the non operated breast, unlike preoperative results. Skin thickness increase is caused by an increased extravascular-extracellular leakage space in the (hypo)dermis and extensive cellular fibrosis, characterized by the loss of consistent pattern in extracellular structures.^16^,^17^ Mean total cutaneous thickness was 2.71 mm in an operated and irradiated breast and 1.35 mm in a non operated/irradiated breast. This difference was significant.^6^ Mean skin thickness of the operated breast following RT in our study was 3.03 mm and 1.64 mm in the non operated/irradiated breast, which is similar to the results of Wratten *et al*. (1.36 mm increase versus 1.39 mm increase following BCS and RT).^6^ These results show that skin thickness increase over 2 mm following BCS and RT is a reliable diagnostic criterion for cutaneous breast oedema.

*Echogenicity of the subcutis* in the operated breast following RT increased in 89.5% of the patients and was significantly higher than prior to surgery, unlike the non operated breast subcutis. Echogenicity of the subcutis of the operated breast following RT was significantly higher than the non operated breast, unlike preoperative results. Increase in echogenicity of the subcutis reflects a hypoechoic area.

Increased echogenicity of the subcutis in the operated breast following RT can be caused by an increased subcutaneous extravascular-extracellular leakage space.^16^,^17^ Parenchymal breast oedema might also be the result of a manifestation of an increased number of perfused microvessels, persistent microvascular leakage, impaired drainage, and loss of architectural integrity of tissue microstructures related to radiation-induced vascular injury.^16^,^17^

Our study results could not be compared with other literature results, because echogenicity depends on the gain used on the HFUS exams, frequency of the probe, tissue characteristics, location on the breast and follow-up time. However, 85% of the patients with BCS and RT had an increase in breast tissue density in the study of Delay *et al*.; and Ronka *et al*. also observed an increased echogenicity.^11^,^12^ Wratten *et al*. described a decrease in echogenicity of the subcutis following BCS and RT.

In our study HFUS settings were standardized, so we can conclude that there was a significant increase of the echogenicity of the subcutis, most likely due to the presence of oedema in the subcutis. Like skin thickness, echogenicity of the subcutis can be a valuable diagnostic criterion, when taking into account standardization of methodology as previously described.

*Visibility of the echogenic line in the subcutis* of the operated breast following RT decreased in all patients and was significantly lower than prior to surgery, unlike the non operated breast. Visibility of the echogenic line in the subcutis of the operated breast following RT was significantly lower than the non operated breast, unlike preoperative results.

These results are similar to the results of Ronka *et al.*^11^ This criterion is subjectively scored, by only one experienced breast radiologist (DB) for all measurements in all patients. Visibility of the echogenic line in the subcutis of an operated breast following RT decreases, because of disturbance of the skin/subcutaneous fat interface due to presence of cutaneous and subcutaneous oedema. Therefore visibility of the deeper echogenic line can also be a useful diagnostic criterion for assessing breast oedema.

*Mean interstitial fluid accumulation* of the operated breast following RT increased in 72.2% of the patients and was significantly higher than prior to surgery, unlike in the non operated breast. Interstitial fluid accumulation of the operated breast following RT was significantly higher than the non operated breast, unlike preoperative results. These results were not significantly higher in the upper quadrants of the operated and irradiated breast. This could be explained by the influence of gravity on the fluid in the breast. Presence of interstitial fluid accumulation in the operated and irradiated breast is an objective HFUS visible entity. It is a valuable diagnostic criterion for presence of interstitial oedema due to fluid leakage in the extracellular interstitial space. Our study results are similar to the results of Ronka *et al.* and Wratten *et al.* who also observed an increased interstitial fluid accumulation in the operated and irradiated breast.^11^,^14^

*Mean elasticity ratio of the subcutis* of the operated breast following RT increased in 88.9% of the patients and was significantly higher than prior to surgery, unlike the non operated breast. Mean elasticity ratio of the subcutis in the operated breast following RT was significantly higher than the non operated breast, unlike preoperative results. These results were not significantly higher for all quadrants of the operated and irradiated breast. Our group compared the strain of the subcutaneous fat tissue with the strain of an elastic gel pad giving an elasticity ratio. Increase in elasticity ratio in the operated and irradiated breast corresponds to more elastic breast tissue in comparison with the preoperative results. An increase in elasticity of the underlying breast tissue could be explained by the increase of fluid in the breast. Our study results could not be compared with other literature results, because to our knowledge, no other research group has used the same methodology.

Elasticity ratio can be a valuable diagnostic criterion, when taking into account standardization of methodology. Although elasticity ratio in the different quadrants of the operated and irradiated breast was higher than baseline measurements and the non operated breast, the difference was not significant. Further research with more patients is necessary to confirm these results.

*Correlations* between different breast oedema criteria were present between mean elasticity ratio and mean visibility of the echogenic line in the operated and irradiated breast only. Wratten *et al.* found a correlation between subjective parenchymal breast oedema and skin thickness and between skin thickness and cutaneous echogenicity.^6^

The absence of other correlations with our new technique could suggest elasticity ratio to be a supplementary criterion for the diagnosis and degree of breast oedema, giving extra information on hydrated breast tissue elasticity. Additionally, making a combination of the four breast oedema criteria, measured with HFUS the incidence of breast oedema was 90.4% following RT, compared to 88.9% with increased elasticity ratio.^11^ The breast oedema definition and our new technique result in equal incidences.

Unlike similar research, we did not found *risk factors* for the increase of parenchymal breast oedema, expressed by increased elasticity ratio in this study, except for bigger preoperative bra cup size.^3^,^6^,^10^,^12^,^13^ All patients had an increased mean elasticity ratio in the operated and irradiated breast, except for patients with preoperative A cup.

Echogenicity and elasticity ratio were obtained from the subcutis and not cutaneous, because breast skin behaves different than the underlying breast tissue and literature showed disagreement on cutaneous measurements.^6^,^11^ Measurements in the inframammary fold of the operated and irradiated breast were not discussed in this manuscript, as well as measurements of the first follow-up (postoperative but prior to RT) for the same reasons.

Our study presents several limitations. Our pilot study counts only 20 patients and a short follow-up. As part of the institution’s surgical management, operated breast cancer patients receive a prescription for ambulatory physical therapy at the time of discharge. However, we did not record the compliance of patients or their receipt of manual lymphatic drainage of the breast during the study, although their beneficial effects on breast oedema could be expected.^34^–^37^ Another limitation of our study is the known lack of reliability of breast oedema criteria measurements. Our study results should be interpreted with these restrictions in mind.

One strength of the study is standardization of HFUS settings, US elastography methodology and measurement protocol, like gain, gauges, etc. by a specialized breast radiologist (DB). Gain was often adjusted to become better clinical results in other studies, but this was not the case in our study.^6^,^11^ Wratten *et al.* concluded that HFUS was not useful in quantifying acute inflammatory changes induced by breast irradiation, but with our standardized approach this was possible.^14^ A second strength of the study is the preoperative measurements to compare with immediate post irradiation measurement. To our knowledge, no other research groups have used preoperative bilateral measurements as baseline results. Another strength of our study is that the trial was conducted in a single institution. All patients were followed by the same team, which ensures that assessments were consistently performed throughout the trial. We believe that the strengths of the study outweigh its limitations and that the results are robust, at least within the current short follow-up time frame.

Because this is a pilot study, the long-term usability of this technique cannot yet be demonstrated; however, this technique seems to be an interesting quantitative objective complement to current breast oedema diagnostic criteria. A follow-up of six months and one year following surgery is scheduled as part of the study, like the time course of parenchymal breast oedema during and following RT was previously described by Wratten *et al*.^6^ Breast oedema incidence peaks at four to six months following treatment and returns to baseline after one year. It will be interesting to see if changes, observed in this analysis are confirmed at later follow-up. Our findings suggest further investigation with more patients and longer follow-up.

We can conclude from our study results that ultrasound elastography is an objective quantitative measurement tool for the diagnosis of parenchymal breast oedema, in combination with other objective diagnostic criteria.

## Figures and Tables

**TABLE 1:** Patient’s characteristics

	**mean**	SD*
Age (years)	58.9	12.1
Body mass index (kg/m^2^)		
Preoperative	26.4	4.6
Postirradiation	26.1	4.7
Time interval (weeks)		
Preoperative – postirradiation	12.7	2.8
Time interval (weeks)		
Operation – start radiation therapy		
Volume of the operated breast (cc)		
Preoperative	961.8	695.8
Postoperative	830.6	558.3
Postirradiation	842.4	599.3

* standard deviation

**Table:** 

	**n**	**Valid %**
Tumours localization in the SEQ**	12	60
Operated side equals dominant side	10	50
Axillary lymph node dissection	5	25
Neoadjuvant chemotherapy	1	5
Anti-hormone therapy	13	65
Chemotherapy	6	30
Irradiation dose		
< 66 Gy	5	25
66 Gy	15	75
Skin toxicity		
Grade 1	15	83,3
Grade 2	2	11,1
Grade 3	1	5,6
Subjective swelling	7	43,8
Preoperative breast cup size		
A	3	15
B	9	45
C	7	35
≥ D	1	5
Preoperative obesity (BMI*** > 30 kg/m^2^)	5	25

** superior external quadrant;

*** body mass index

**TABLE 2:** Skin thickness (mm) in the different quadrants of both breasts (operated and non operated) at baseline (pre) and follow-up (post)

	**Operated – pre**	**Non operated – pre**	**Operated – post**	**Non operated – post**
Mean	1.66 (.28)	1.65 (.26)	3.03 (1.28)	1.62 (.22)
SIQ	1.73 (.32)	1.72 (.28)	2.87 (1.05)	1.68 (.27)
IIQ	1.7 (.28)	1.69 (.29)	3.43 (1.9)	1.75 (.32)
IEQ	1.65 (.33)	1.64 (.29)	3.12 (1.34)	1.54 (.27)
SEQ	1.56 (.30)	1.55 (.26)	2.7 (1.10)	1.52 (.25)

SIQ, upper inner quadrant; IIQ, lower inner quadrant; IEQ, lower outer quadrant and SEQ, upper outer quadrant

**TABLE 3:** Echogenicity of the subcutis in the different quadrants of both breasts (operated and non operated) at baseline (pre) and follow-up (post)

	**Operated – pre**	**Non operated – pre**	**Operated – post**	**Non operated – post**
Mean	110.3 (18.1)	113.9 (13.5)	124.2 (19.9)	106.7 (16.6)
SIQ	110.3 (20.1)	116.9 (20.6)	127.9 (19.1)	110.1 (21.3)
IIQ	105.6 (18.4)	108.8 (21.2)	121.1 (19.9)	99.4 (17.1)
IEQ	111.3 (22.5)	114.1 (32.3)	129 (21.9)	110 (17.7)
SEQ	114 (21)	111.3 (21.5)	122.7 (28.6)	107.4 (19.3)

SIQ, upper inner quadrant; IIQ, lower inner quadrant; IEQ, lower outer quadrant and SEQ, upper outer quadrant

**TABLE 4:** US Elasticity ratio of the subcutis in the different quadrants of both breasts (operated and non operated) at baseline (pre) and follow-up (post)

	**Operated - pre**	**Non operated - pre**	**Operated - post**	**Non operated - post**
Mean	2.78 (1.66)	2.85 (1.47)	4.06 (1.31)	3.05 (1.25)
SIQ	2.77 (1.65)	3.38 (2.63)	4.08 (2.22)	3.35 (2.08)
IIQ	2.68 (1.65)	2.77 (1.76)	3.63 (1.77)	3.06 (1.87)
IEQ	3.09 (3.14)	2.44 (1.3)	3.92 (2.60)	2.92 (1.4)
SEQ	2.57 (1.18)	2.82 (2.14)	3.34 (2.46)	2.9 (1.48)

SIQ, upper inner quadrant; IIQ, lower inner quadrant; IEQ, lower outer quadrant and SEQ, upper outer quadrant
